# Vector-delivered artificial miRNA effectively inhibits Porcine epidemic diarrhea virus replication

**DOI:** 10.1186/s12985-023-02129-5

**Published:** 2023-07-24

**Authors:** Tingfan Zhu, Jinhan Qian, Zijun Shen, Hongxia Shao, Kun Qian, Wenjie Jin, Aijian Qin

**Affiliations:** 1 College of Veterinary Medicine, Yangzhou University, Yangzhou, 225009 Jiangsu Province China; 2 Jiangsu Co-Innovation Center for Prevention and Control of Important Animal Infectious Diseases and Zoonosis, Yangzhou, 225009 Jiangsu Province China; 3 Ministry of Education Key Laboratory of Poultry Preventive Medicine, Yangzhou, 225009 Jiangsu Province China

**Keywords:** Porcine epidemic diarrhea virus, RNAi, Artificial microRNA (amiRNA)

## Abstract

**Background:**

Porcine epidemic diarrhea virus (PEDV) is an α-coronavirus that causes highly contagious intestinal infectious disease, involving clinically characterized by diarrhea, dehydration, vomiting, and high mortality to suckling piglets. As a strategy for antiviral therapy, artificial microRNA (amiRNA) mediated suppression of viral replication has recently become increasingly important. In this study, we evaluated the advantages of using an amiRNA vector against PEDV.

**Methods:**

In this study, we evaluated the advantages of using an amiRNA vector against PEDV. We designed two single amiRNA sequences for different conserved sequences of the PEDV S and N genes, and tested their inhibitory effects on PEDV in Vero cells.

**Results:**

It was obvious from the CCK-8 results that the transient transfection of amiRNA was non-toxic to the cells. In addition, our results showed that the transient expression of two amiRNAs (amiRNA-349 and amiRNA-1447) significantly reduced the expression of viral RNA and protein in the cells. The TCID_50_ results showed that the release of virus particles into the culture supernatant was significantly reduced, with an effect as high as 90%. To avoid virus mutation escape, the above two single amiRNA sequences were tandem in this study (amiRNA-349 + 1447), enabling a single microRNA to be expressed simultaneously. The real-time PCR and Western blot results showed that the inhibitory effect was significantly enhanced in each of the different time periods. The TCID_50_ results showed that the release of virus particles in the culture supernatant was significantly reduced at the different time periods.

**Conclusions:**

In summary, these results suggest that an RNAi based on amiRNA targeting the conserved region of the virus is an effective method to improve PEDV nucleic acid inhibitors and provide a novel treatment strategy for PEDV infection.

## Introduction

Porcine Epidemic Diarrhea Virus (PEDV) is a non-segmented single-stranded plus-stranded cystic RNA virus that belongs to the order *Nidovirales*, family *Coronaviridae*, and α-coronavirus genus [1]. PEDV is transmitted via the fecal–oral route and nasal inhalation. Once in the piglets, the virus begins to destroy the cells of the gut, depriving the intestine of its ability to digest and absorb nutrients from milk and food [2]. Moreover, PEDV primarily causes acute diarrhea, vomiting, dehydration, and death in young piglets. Most of the infected piglets die within 7 days after birth, and the mortality rate of young piglets was as high as 100%, which causes substantial losses to the pig industry [3, 4]. The PEDV genome encodes four structural proteins: S (spike protein), M (membrane protein), E (envelope protein), and N (nucleocapsid protein). The N protein encoded by the N gene is a phosphorylated nucleocapsid protein and the only phosphorylated protein among the known coronavirus structural proteins. N is a highly conserved phosphoprotein, which exhibits only a few point mutations in the different strains. The N protein has been associated with multiple functions in the viral life cycle, including regulation of the viral RNA synthesis, the packaging of the viral RNA into helical nucleocapsids, and virion assembly [5]. The S gene encodes fibrin with a total length of 4,152 nt. The S protein encoded by the S gene is a membrane glycoprotein expressed on the virion surface, and is approximately 150 kDa [6]. The PEDV S protein exhibits a high degree of genetic diversity and plays a pivotal role in mediating viral entry, inducing neutralizing antibodies and viral virulence in vivo [7].

RNA interference (RNAi) is a process in which small interfering RNAs (siRNAs) or endogenous microRNAs (miRNAs) with protein partners regulate cellular gene expression at the post-transcriptional level by the degradation of complementary transcripts and/or inhibition of translation [8, 9]. The application of RNA interference technology has produced novel discoveries in the field of antiviral therapy and now shows potential application prospects. Since 2008, some scholars have used RNAi technology to treat HBV. A large number of experimental studies in vivo and in vitro have demonstrated that the use of siRNA to control the expression and replication of HBV is efficient and feasible [10]. However, several viruses can escape RNAi under the pressure of treatment selection through the mutation of target gene sequences and generation of inhibitory factors. Moreover, the therapeutic effect of RNAi targeting viruses may be reduced by the viral mutations, or even lose the therapeutic effect. Compared with siRNA, miRNA only needs to be partially bound to target genes to have an effect, so it can largely avoid the failure of RNAi therapy caused by viral mutation [11, 12]. Since miRNA and its target do not require strict base complementary pairing, it may be more difficult for viral mutations to escape miRNA-specific silencing, which will be more advantageous for the treatment of mutation-prone viruses (e.g., PEDV and HIV) diseases [13]. However, Su et al. reported that the PEDV virus exhibits strong variability, and PEDV mutant strains have become the dominant PEDV epidemic strains in China. Therefore, miRNA has substantial advantages for the treatment of PEDV infection. The study of miRNA function may help to propose new methods for the treatment of viral diseases. In addition, no obvious off-target effects or side effects have been identified in miRNA-mediated RNAi. MiRNA has emerged as a target candidate for the development of novel RNA interference gene therapy approaches [14]. Therefore, this study designed and synthesized artificial microRNAs that target the PEDV N and S genes, constructed artificial miRNA expression vectors, and transfected Vero cells to observe the inhibitory effect of artificial miRNA on the transcription of genes from target cells, and further observe the effect on PEDV proliferation.

Research into PEDV gene therapy using synthetic artificial microRNAs has not been previously reported. Moreover, related studies have shown that the miRNA expression framework produces exogenous miRNAs that can specifically degrade target genes that have shown favorable application prospects.

## Materials and methods

### Cells and viruses

African green monkey kidney cells (Vero cells) were preserved by the Jiangsu Co-innovation Center for Prevention and Control of Important Animal Infectious Diseases and Zoonosis. The cells were cultured in a disposable 25 cm^2^ square flask with Dulbecco's Modified Eagle Media (DMEM) medium containing 5% fetal bovine serum and 1% Penicillin–streptomycin (double antibiotics). The culture conditions were 37 °C and 5% CO_2_ in an incubator. PEDV strains were isolated and preserved by Jiangsu Co-innovation Center for Prevention and Control of Important Animal Infectious Diseases and Zoonosis, Yangzhou University. PEDV was subcultured using standard virus adsorption techniques. The viral titers were measured in Vero cells with a TCID_50_ and stored at − 80 °C until further use.

### Selection and design of PEDV interference area

Bioinformatics analyses were used to perform a multiple sequence homology alignment on existing PEDV N and S gene sequences on NCBI, and the conserved sequences were identified as the miRNA target regions of PEDV by a combination analysis (Table 1). AmiRNA interference region selection: the Invitrogen BLOCK-IT™ RNAi (http://www.invitrogen.com/rnai) expression onlinesearch engine design software was applied. According to the optimization principle, sequences with a content of 40–55% were selected for PEDV N and S gene conserved regions, and the scoring principle should be above 4.5 points. The selected sequences were subjected to an online homologous analysis with the porcine source gene sequences in GenBank to minimize the potential non-specific target effect, and two corresponding specific expression sequences were designed. The negative control was provided by Invitrogen, and the single chain oligonucleotide (Oligo) was synthesized by Nanjing Qingke Biotechnology. The plasmid contains an insert that can form hairpin structures and be processed into mature miRNAs; however, it is not expected to target any known vertebrate genes, and the synthesized sequences are all 64 bp in length.

**Table 1:** Oligonucleotide sequences designed for amiRNA construction

amiRNA	Sequences (5′–3′)
amiRNA-349	5′-TGCTGAGTACGAGTCCTATAACGGAGGTTTTGGCCACTGACTGACCTCCGTTAGGACTCGTACT -3′
	5′-CCTGAGTACGAGTCCTAACGGAGGTCAGTCAGTGGCCAAAACCTCCGTTATAGGACTCGTACTC -3′
amiRNA-1447	5′-TGCTGAGTACGAGTCCTATAACGGAGGTTTTGGCCACTGACTGACCTCCGTTAGGACTCGTACT -3′
	5′-CCTGAGTACGAGTCCTAACGGAGGTCAGTCAGTGGCCAAAACCTCCGTTATAGGACTCGTACTC -3′

Two sequences were designed using Invitrogen Block ^iT^ RNAi Designer targeting sequences of the N and S regions of PEDV.

### Construction of miRNA expression vectors, as well as the construction, transformation, and purification of the plasmid and identification of the recombinant plasmid

Each pair of single-chain oligonucleotides was annealed, and the reaction system was: positive DNA oligo (200 μm) 5 μL, negative DNA oligo (200 μm) 5 μL, 10× Annealing Buffer 2 μL (100 mM Tris–HCl pH 8.0, 500 mM NaCl, 10 mM EDTA), and 8 μL deionized water. The reaction system was incubated at 94 °C for 5 min and then slowly cooled to room temperature for annealing. The double-stranded oligonucleotides were cloned into the pcDNA™6.2-GW/ EmGFP-miR expression vector to construct the eukaryotic expression plasmid, and the ligation reaction product was transformed into the TOP10 competent cells. Monoclonal colonies were selected for amplification and culture, and the plasmid was extracted. The restriction analysis was performed by *BamH*I and *Xho*I, and the correct plasmid was identified by enzyme digestion. EmGFP forward sequencing primers and miRNA reverse sequencing primers were sent to Nanjing Qingke Biotechnology for sequencing. The selected positive recombinant plasmids were further confirmed by sequencing, and the correctly sequenced plasmids were termed amiRNA-349 and amiRNA-1447, respectively.

### Optimization of cell transfection efficiency

The day prior to transfection, Vero cells were digested and seeded into six-well cell plates at a density of 4 × 10^5^ cells per well. When the cell density reached 70–80%. The ratio of plasmid amiRNA-349: Lipofectamine™3000 was 1:0.75–1:1.5, and the plasmid dose was 4 μg, 5 μg, and 6 μg, respectively. Lipofectamine™3000 was used as the transfection reagent for the plasmid transfection. After a 48 h culture for transfection efficiency was observed and photographed.

### Cytotoxicity assay

Cell Counting Kit-8 (CCK-8) (Vazyme, China) was performed in accordance with the manufacturer's instructions. Vero cells were digested and added to 96 well plates at a density of 2 × 10^4^ cells per well and transfected with 100–800 ng/well of plasmids expressing amiRNA in three replicates for each concentration. Cell viability was detected using a Cell Counting Kit-8 (CCK-8) assay after Vero cells were transfected with amiRNAs for 48 h.

### CCK-8-mediated detection of cytotoxicity

Under optimized transfection conditions, miRNA expression vectors were transfected into Vero cells. The negative control and no-load control were established at the same time. After normal culture, PEDV strains with 0.01 MOI were inoculated in each well. Cell viability was measured 48 h after PEDV infection using CCK-8 Cell Counting Kit according to the manufacturer's instructions. The absorbance values of amiRNA-treated and virus-infected cells were compared with those uninfected samples without transfection reagents and amiRNA.

### Detection of the viral titer

The cell culture, plasmid transfection, and PEDV infection conditions were performed as described above. The cell supernatants were collected at 24 h, 48 h, and 72 h after viral infection, and their TCID_50_ was determined using the Reed-Muench method.

### Real time RT-PCR assay for the detection of PEDV RNA

The cell culture, plasmid transfection, and PEDV infection conditions were performed as described above. The cells were collected at 24 h, 48 h, and 72 h after infection and the total RNA was extracted. Using the 18 s gene as an internal reference, PEDV N gene transcription was detected by relative quantitative RT-PCR according to the instructions of the ChamQ Universal SYBR qPCR Master Mix (Vazyme, China). The positive and reverse primer sequences of the internal reference were as follows:forward primer (18 s F), 5′–TCAGATACCGTCGTAGTTCC–3′;reverse primer (18 s R), 5′–TTCCGTCAATTCCTTTAAGTT–3′.

The specific primer sequences of N gene were as follows:forward primer (PEDV-N F), 5′–CGATGATCTGGTGGCTGCTGTC–3′;reverse primer (PEDV-N R), 5′–TTCCTGCTTAGGCTTCTGCTGTTG–3′.

The 20 μL PCR reaction system consisted of ChamQ Universal SYBR qPCR Master Mix (2×) 10 μL, forward and reverse primers 0.4 μL, reverse transcription cDNA template 2 μL, and ddH_2_O_2_ 7.2 μL. Amplification procedure 95 °C, pre-denaturation 30 s; 95 °C for 5 s, 60 °C for 34 s, for 40 cycles; 95 °C for 15 s, 60 °C for 60 s, and 95 °C for 15 s. PCR products were used to analyze the relative expression of the PEDV N gene in each group using a 7500 Real-time PCR System software. At the same time, the 18 s gene was set as the internal reference gene, and three replicates were performed in each group.

### Western Blot analysis

The cell culture, plasmid transfection, and PEDV infection conditions were performed as described above. The Vero cells were collected PI24h, 48 h, and 72 h, and RIPA (Beyotime, China) was added and placed on ice. The cells were shaken three times once every 10 min, and centrifuged. The supernatant was collected and the α-tubulin protein was used as an internal reference. The cell lysates were dissolved on 12.5% polyacrylamide gel and then electroimprinted on an NC membrane. The membrane was sealed with 5% BSA at 4 °C overnight. The membrane was then incubated with an anti-PEDV-specific polyclonal antibody and an α-tubulin (Cell Signaling Technology, USA) of PEDV was used as an internal sample control.

### Chaining of amiRNAs in a single expression construct

This study uses a pcDNA™ 6.2-GW/EmGFP-miR expression vector which permits the co-cistronic expression of multiple amiRNAs in a single expression construct. Briefly, to construct an expression vector which simultaneously expresses two amiRNA, and the donor plasmid is first excised with *BamH*I/*Xho*I restriction digestion enzymes to release the insert. The insert was ligated into another amiRNA vector backbone, which is predigested with *Bgl*II/*Xho*I enzymes. A single vector constructs generated by chaining of amiRNA-349 and amiRNA-1447, termed amiRNA-349 + 1447. The effect of concatenated amiRNA-349 + 1447 in PEDV replication inhibition was studied by TCID_50_, relative qRT-PCR, and Western blot analysis as mentioned above.

### Statistical analysis

Results were graphed, with error bars indicating the standard deviation. Statistical analyses were done with Prism 8.4.3(GraphPad Software), and statistical significance was determined using Student’s *t* test or one-way analysis of variance (*P < 0.05; **P < 0.01; ***P < 0.001; ****P < 0.0001).

## Results

### Design and identification of recombinant amiRNA plasmids

All definitions of amiRNAs used in this study are listed in Table 1, which are termed amiRNA-349 and amirRNA-1447. Two effective amiRNAs targeting PEDV were designed, and their target sequences were highly conserved among all PEDV reference sequences in GenBank. Two amiRNAs were synthesized and cloned into a pcDNA™6.2-GW/EmGFP-miR vector. Next, positive clones were screened through spectinomycin resistance and correctness was confirmed by DNA sequencing.

### Transfection efficiency exploration

**Fig. 1:**
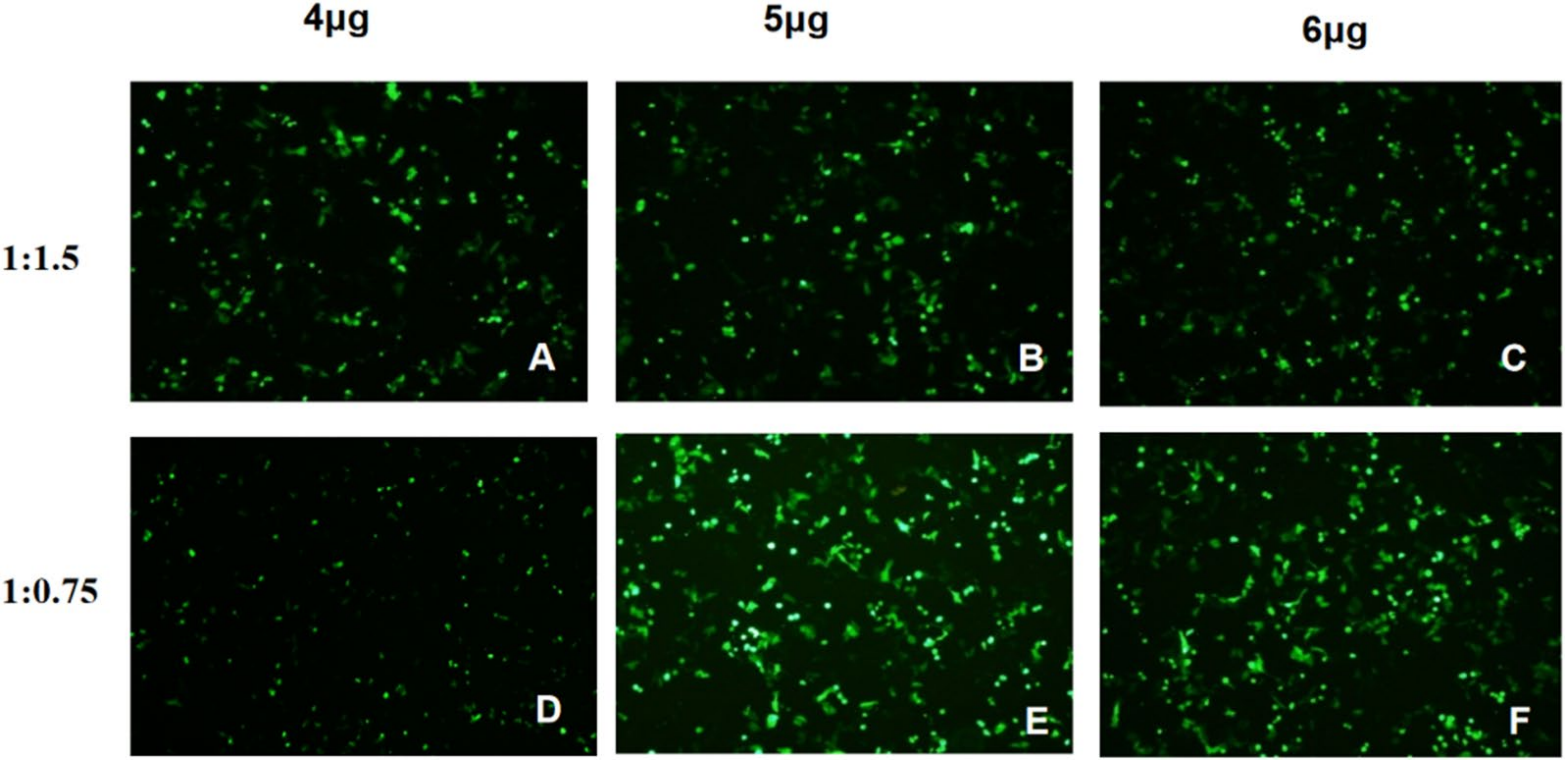
The ratio of plasmid amiRNA-349: Lipofectamine™3000 was 1:0.75, 1:1.5, the plasmid mass was 4 μg, 5 μg, and 6 μg, respectively, with a cell density of 4 × 10^5^. The experiments were carried out in a six-well plate, and the fluorescence renderings of plasmid transfection under different transfection ratios were filmed. The proportion with the highest transfection efficiency was selected for subsequent experiments. The fluorescence microscope images of Vero cells were observed 48 h after transfection, green fluorescence displayed the level of EmGFP expression, and amiRNAs showed the transfection efficiency. **A** amiRNA-349: Lipofectamine3000 was 1:1.5, the plasmid mass was 4 μg. **B** amiRNA-349: Lipofectamine3000 was 1:1.5, the plasmid mass was 5 μg. **C** amiRNA-349: Lipofectamine3000 was 1:1.5, the plasmid mass was 6 μg. **D** amiRNA-349: Lipofectamine3000 was 1:0.75, the plasmid mass was 4 μg. **E** amiRNA-349: Lipofectamine3000 was 1: 0.75, the plasmid mass was 5 μg. **F** amiRNA-349: Lipofectamine3000 was 1: 0.75, the plasmid mass was 6 μg

To achieve a higher transfection efficiency, the ratio of amiRNA-349: Lipofectamine™3000 was 1:0.75–1:1.5, and the plasmid mass was 4 μg, 5 μg, and 6 μg, respectively. The experiment was carried out in a 6-well plate cell density was 4 × 10^5^/mL and the fluorescence effect diagram of plasmid transfection was filmed at different transfection ratios (Fig. 1). The results showed that the plasmid amiRNA-349: Lipofectamine™ 3000 ratio was 1: 0.75, and the transfection efficiency was the highest when the plasmid was transfected at 5 μg. Thus, all subsequent experiments were carried out at this ratio.

### AmiRNA plasmids did not affect cell viability

**Fig. 2:**
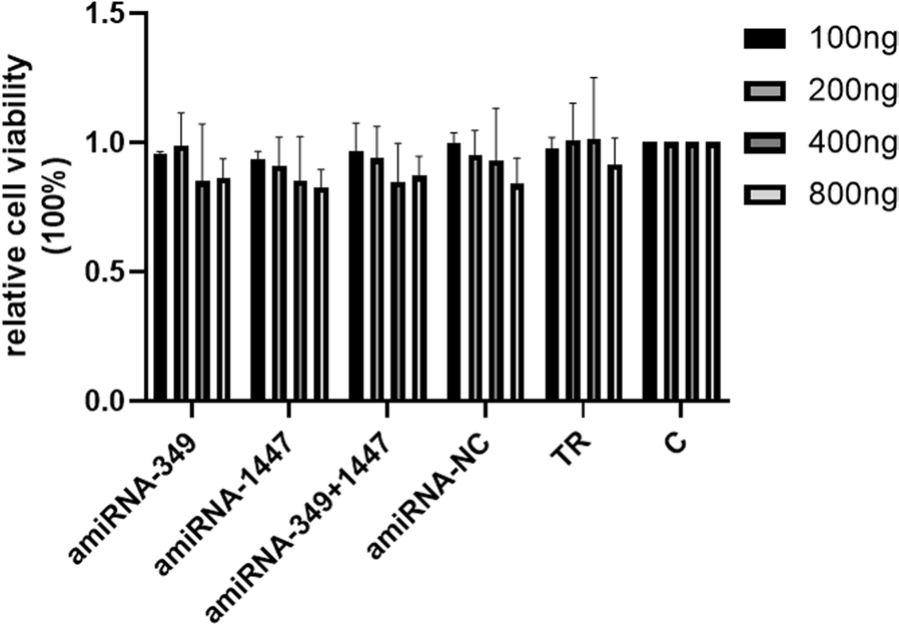
AmiRNA treatment did not affect cell viability. Vero cells were grown in 96 well plates and transfected with 100–800 ng/well of plasmids expressing amiRNA in three replicates for each concentration. Cell viability was detected using cell counting kit-8 (CCK-8) assays after transfecting Vero cells with amiRNAs for 48 h. Absorption at 450 nm was recorded and expressed as the percentage of relative cell viability. Values are expressed as the mean ± SD (n = 3). Significant differences were assessed by a Student’s *t* test; ns, no significant difference compared to the control; *P* > 0.05

To test the cytotoxicity of amiRNA transfected at a dose of 5 µg, Vero cells were inoculated into 96-well plates. When the cells reached 70–80% confluency, Lipofectamine 3000 (Invitrogen, USA) was used to transfect the cells with amiRNA-349, amiRNA-1447, and amiRNA-NC. At the same time, three replicates were established for the cell control and single transfection reagent control. At 48 h post-transfection, the CCK-8 solution (10 µL) was added to the well and the cells were incubated at 37 °C for 2 h. A microtitration plate reader (Bio-TeK, USA) measured the absorbance at 450 nm. The results showed that transfection with 5 µg of the plasmid did not exhibit significant cytotoxicity to Vero cells (Fig. 2).

### Significant antiviral activity of transiently transfected single-amiRNAs

**Fig. 3:**
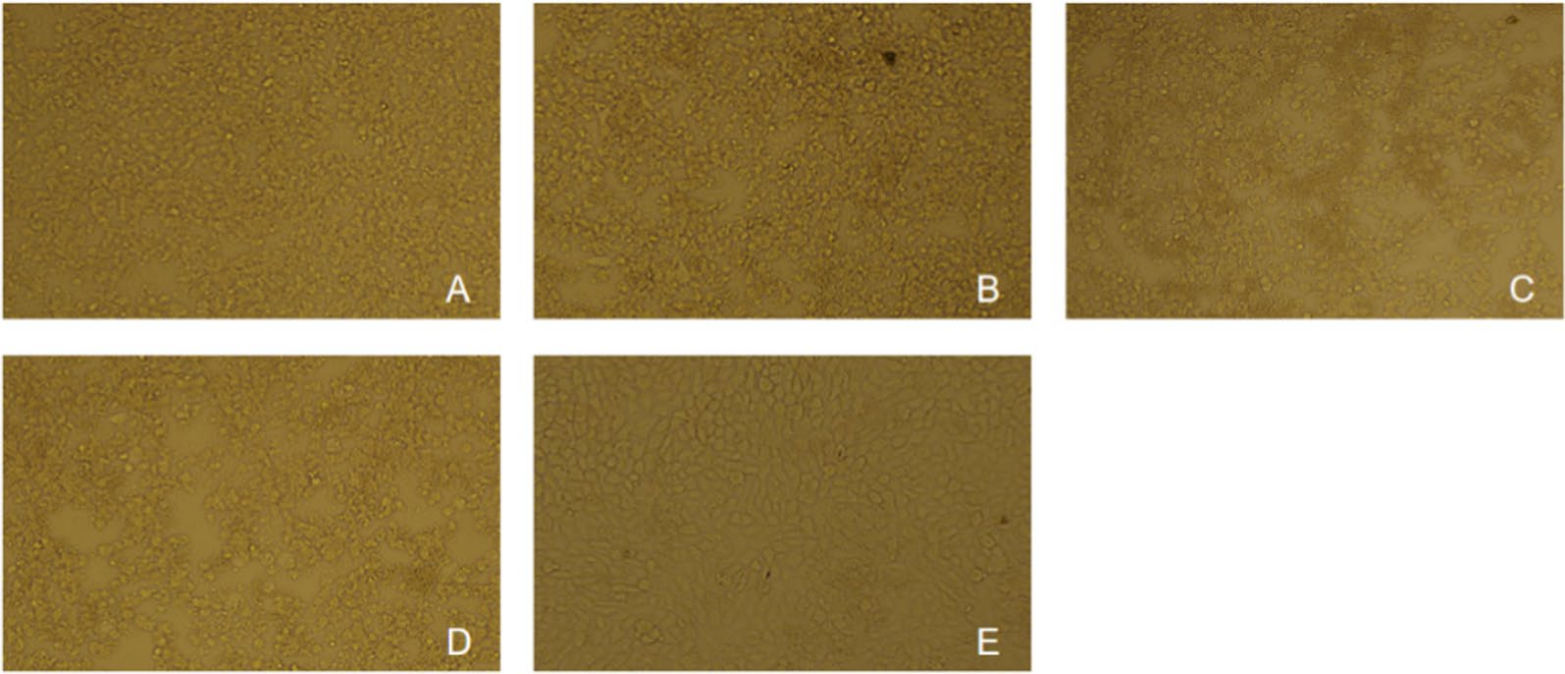
CPE analysis of PEDV on Vero cells transfected with amiRNA-349, amiRNA-1447, or amiRNA-NC. Cells were challenged with PEDV at 0.01 MOI and incubated for 48 h, Magnification: ×10. Microscopic images of Vero cells showing morphology of Vero cells at 48 hpi after respective treatment. Data shown here is representative of one of the three experimental repeats. **A** amiRNA-349, **B** amiRNA-1447, **C** amiRNA-NC, **D** virus control, and **E** normal cell control

**Fig. 4:**
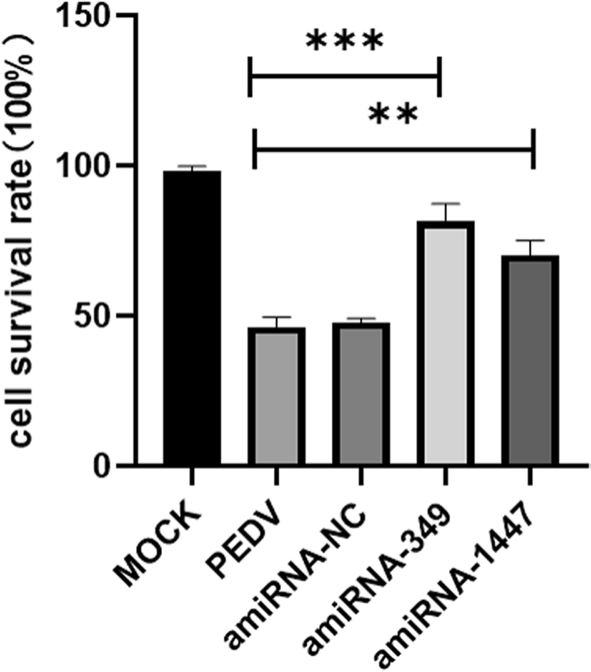
Comparison of the cell survival rates at 48 h post-infection among the transfected cells

To identify whether amiRNA could prevent CPE-induced PEDV, Vero cells were selected as the host cells to evaluate the antiviral effect of amiRNAs since they had a high PEDV infection rate and relatively high amiRNA transfection rate. Both groups included amiRNA-349, amiRNA-1447, amiRNA-NC, virus infection control (PEDV contained without amiRNA and transfection reagents), and normal cell control. Based on the CPE, we filmed the cell morphology of Vero cells infected with PEDV following amiRNA treatment (Fig. 3). A CCK-8 assay was used to evaluate the protective effect of each amiRNA on the viability of infected Vero cells at 48 h after PEDV infection (Fig. 4). These results indicated that the amiRNA treatment group had a significant protective effect on cell survival.

**Fig. 5:**
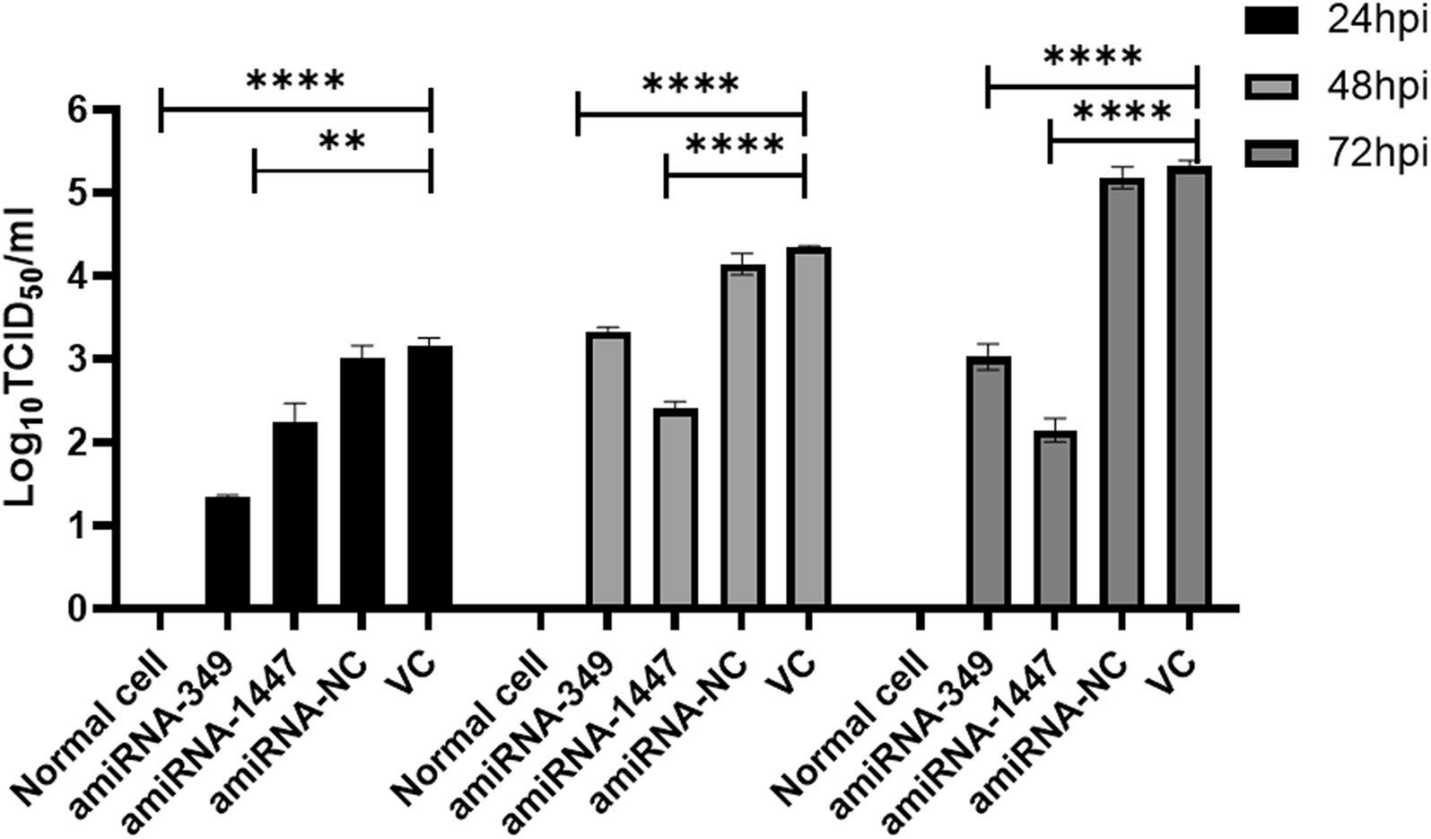
Comparison of the PEDV titer in the cell supernatants harvested at 24, 48, 72 h post infection. **P < 0.01; ****P < 0.0001. All data represent the mean and standard deviation of three independent experiments each performed in triplicate

**Fig. 6:**
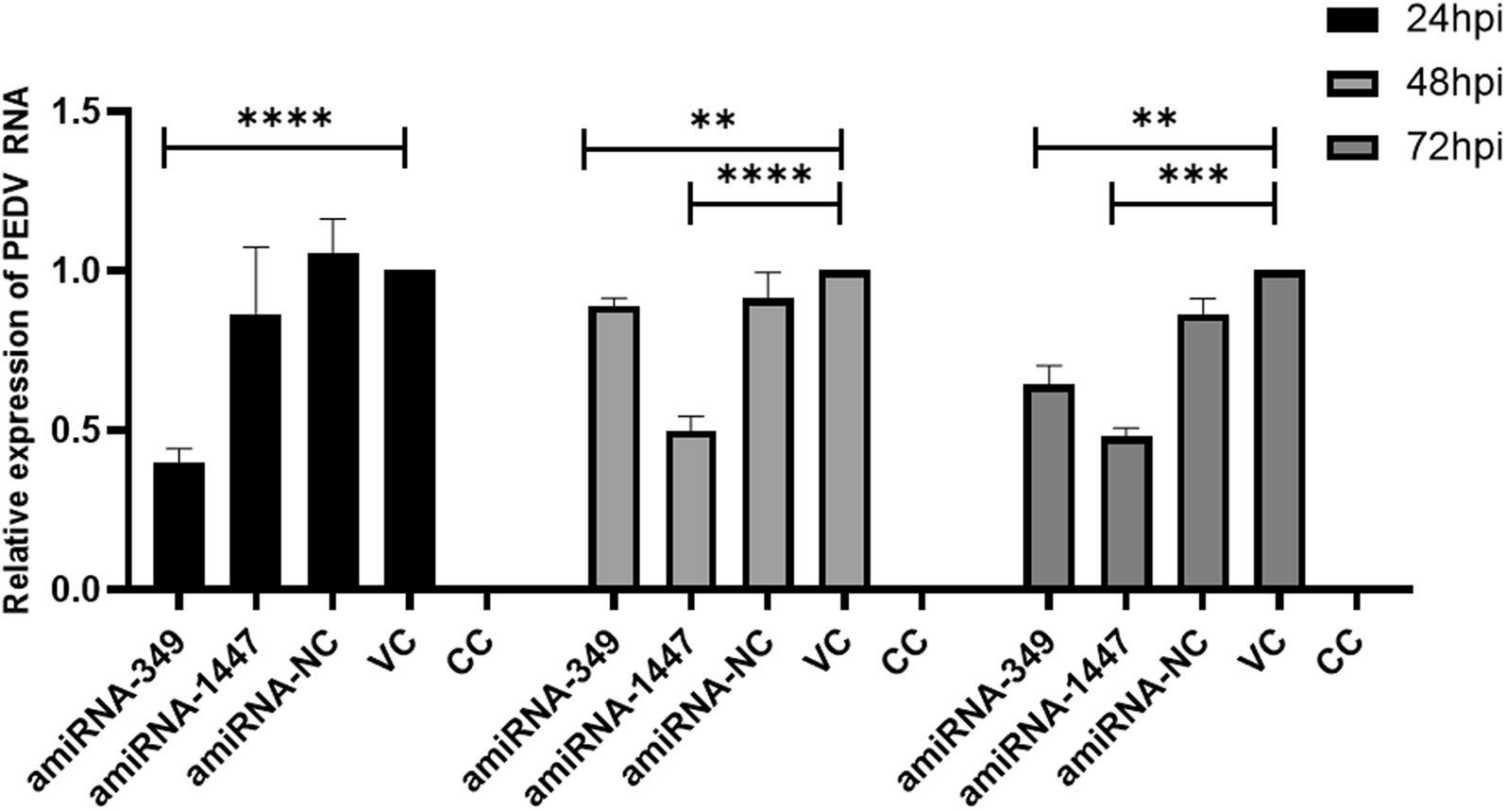
Relative expression of PEDV genomic RNA. Identification of relative PEDV RNA copy number by qRT-PCR. Vero cells were treated with artificial miRNA and infected with 0.01 MOI PEDV. Cells were harvested at 24, 48 and 72 hpi and RNA extraction was performed. qRT-PCR was carried out with specific primers for PEDV (**P < 0.01; ***P < 0.001; ****P < 0.0001)

**Fig. 7:**
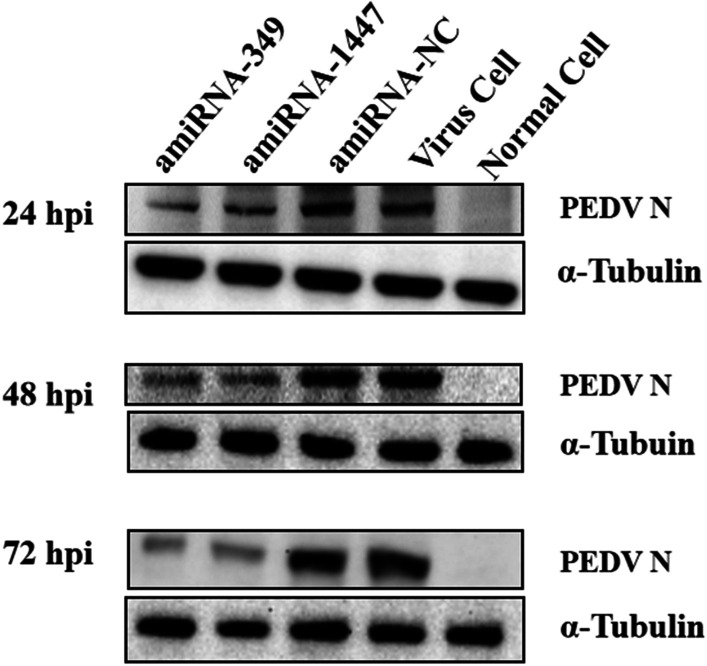
Western blots showing the amount of viral N protein expression in Vero cells lysates following treatment with different amiRNAs. Vero cells were treated with artificial miRNAs and infected with 0.01 MOI PEDV. The total cell lysates containing 40 μg protein per sample was loaded. α-tubulin served as the internal quantity and loading control

The supernatant of the cells treated with amiRNA was collected at 24 h, 48 h, and 72 h following PEDV infection to determine the TCID_50_ (Fig. 5). The experimental results showed that the TCID_50_ in the amiRNA-treated group was significantly lower than that in the amiRNA-NC and VC groups. The collected cells were verified by real-time PCR, and the results showed that the PEDV mRNA in the amiRNA- treated group was significantly decreased compared with that in the amiRNA-NC and VC groups (Fig. 6). The Western blotting results showed that the PEDV N protein expression in the amiRNA- treated group was significantly decreased compared with that in the amiRNA-NC and VC groups (Fig. 7). All of the above results are consistent.

### Significant antiviral activity of transiently transfected concatenated amiRNA

**Fig. 8:**
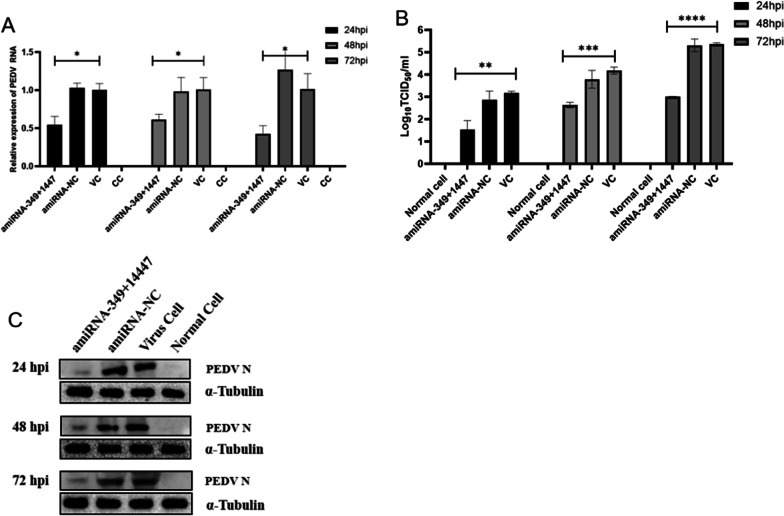
The combination of amiRNA-349 and amiRNA-1447. **A** A reduction of the PEDV RNA copy number as assessed by QRT-PCR with PEDV specific primers. *P < 0.05. **B** Comparison of PEDV titer in the cell supernatant harvested at 24, 48, and 72 h post-infection. **P < 0.01; ***P < 0.001; ****P < 0.0001. **C** Western blot showing the amount of the viral N protein in Vero cell lysates following treatment with different amiRNAs. The total cell lysate containing 40 mg protein per sample was loaded. α-tubulin served as an internal quantity and loading control

As assessed by real-time PCR, TCID_50_ and Western blotting techniques (Fig. 8), the combination of the two optimal amiRNA-349 + 1447 could significantly inhibit PEDV replication. In general, amiRNA-349 + 1447 inhibition was higher than that of amiRNA-349 and amiRNA-1447.

## Discussion

Porcine epidemic diarrhea (PED) is a highly infectious intestinal infection caused by PEDV. Since 2010, large-scale PED outbreaks have been reported in China, with a high mortality rate in suckling piglets [15, 16]. The rapidly emerging PED outbreaks have spread throughout the world, as cases have been reported in Vietnam and Thailand [17], South Korea [18], Japan [17], the United States [19], and Italy [4]. It is even widely believed that the PED variant will be one of the most serious diarrhea diseases affecting piglets over the next few years. Similar to most viral diseases, there is no specific drug for PED prevention and control, and primarily depends on vaccination. For decades, both domestic and foreign pig disease researchers have developed PED inactivated vaccines, live vaccines, and genetic engineering vaccines [20–23]; however, there is no specific drug for PED in the market, and the research and development of its vaccine is less than ideal [24]. Therefore, it is necessary and urgent to identify novel therapeutic strategies to treat PEDV infection. To date, amiRNAs have been used to suppress the replication of viruses, including Chikungunya virus [25], hepatitis B [26], Venezuelan equine encephalitis virus [27], dengue virus [28], West Nile virus [29], and Japanese encephalitis virus [29]. To date, there has been no published data available for the inhibition of PEDV by vector delivered amiRNA. This is the first study describing the inhibition of PEDV infection by vector-delivered amiRNA in Vero cells. Studies have demonstrated that artificial miRNA-based therapeutics have been shown to be less toxic as it is driven by RNA Pol II promoters that control the expression of amiRNAs, which leads to lower toxicity in cells as compared to conventional shRNAs.

In this study, we designed amiRNA-349 and amiRNA-1447 to target the conserved regions of PEDV N and S genes, respectively. The effect of RNAi based on amiRNA inhibition of PEDV replication and expression in vitro was evaluated. CPE and CCK-8 were used to study whether amiRNA could inhibit PEDV replication in vitro. The results showed that the cytopathic changes in the amiRNA-349 and amiRNA-1447 groups were substantially lower, and the cell survival rate was better than that of VC and amiRNA-NC group at 48 h post-infection. Real-time PCR, Western blot, and TCID_50_ were used to analyze whether amiRNA could inhibit PEDV replication, RNA transcription, and protein expression. The results showed that the inhibitory effect of amiRNA-1447 on PEDV was greater than that of amiRNA-349 at 48 h and 72 h, whereas the inhibitory effect of amiRNA-1447 on PEDV was at low levels at 24 h. We predict that this finding may be due to difference in the function of the targeted gene in viral replication, resulting in the time-varying effect of amiRNA.

Knockdown efficiency of natural miRNA can be increased by simultaneous targeting with the multiple genes. An miRNA-based vector expression system has one benefit over conventional shRNA vector in its capability to express multiple artificial miRNA in a single construct. We also carried out the combination of amiRNA-349 and amiRNA-1447 and then found that concatenated amiRNA-349 + 1447 is able to provide better protection than amiRNA-349 and amiRNA-1447. Overall, this study demonstrated that targeting the N and S genes is essential to amiRNA-based RNAi and offers a smart target for the development of a nucleic acid-based PEDV inhibitor.

This is the first report to demonstrate the successful application of vector-delivered amiRNA for the inhibition of PEDV replication. The efficient in vitro inhibition of PEDV replication exhibited by amiRNA makes it a promising candidate for the development of anti-PEDV therapeutics. Moreover, targeting conserved sequences across all PEDV genotypes makes it an excellent candidate. Combinations with RNAi will provide further insight into the rational selection of combination regimes in the future; however, the efficacy of these amiRNAs remains to be assessed in vivo. Although our knowledge of RNAi therapeutics and combination therapy has increased in recent years, several important issues must be studied carefully (e.g., methods of safe delivery, immune response, and dose of the various combinations) if this knowledge is to be further extended for the development of anti-PEDV therapeutics.

## Conclusions

In summary, our data suggest that an RNAi based on amiRNA targeting the conserved region of the virus is an effective method to improve PEDV nucleic acid inhibitors and provide a novel treatment strategy for PEDV infection.

## Data Availability

The datasets used during the current study are available from the corresponding author upon reasonable request. The datasets generated during the current study are available in the GenBank repository.

## Abbreviations

PED: Porcine epidemic diarrhea
PEDV: Porcine epidemic diarrhea virus
amiRNA: Artificial microRNA
RNA: Ribonucleic acid
S: Spike protein
E: Envelope protein
N: Nucleocapsid protein
M: Membrane protein
RNAi: RNA interference
siRNAs: Small interfering RNAs
DMEM: Dulbecco's Modified Eagle Media
MEGA: Molecular Evolutionary Genetics Analysis
CCK-8: Cell Counting Kit-8
